# Three new species of *Lathrobium* Gravenhorst (Coleoptera, Staphylinidae, Paederinae) from Sichuan, Southwest China

**DOI:** 10.3897/zookeys.205.3148

**Published:** 2012-07-04

**Authors:** Zhong Peng, Li-Zhen Li, Mei-Jun Zhao

**Affiliations:** 1 Department of Biology, College of Life and Environmental Sciences, Shanghai Normal University, Shanghai, 200234, P. R. China

**Keywords:** Coleoptera, Staphylinidae, taxonomy, *Lathrobium*, new species, key to species, checklist, Sichuan, China

## Abstract

Three new species of the genus *Lathrobium* Gravenhorst, 1802 from Sichuan Province, Southwest China are described and illustrated: *Lathrobium acutissimum* **sp. n.**, *Lathrobium hailuogouense* **sp. n.** and *Lathrobium labahense* **sp. n.** A checklist of Chinese *Lathrobium* is provided.

## Introduction

Up to today, 57 species of the genus *Lathrobium* Gravenhorst, 1802 have been reported from China. The records of four species are doubtful and the presence of the remaining 53 species has been confirmed in the past (Assing 2009, 2010a, b; Chen et al. 2005a, b; Hua 2002; Li 1992; Li and Chen 1990, 1993; Peng and Zhao 2012a, b; Ryvkin 2011; Schülke 2002; Smetana 2004; Watanabe 1997, 1998, 1999a, b, 2005, 2011; Watanabe and Xiao 1994, 1996, 1997, 2000).

Only one species, *Lathrobium watanabei* Schülke, 2002, had been known from the vast Sichuan Province, China. In the neighbouring provinces, seventeen species have been recorded from Yunnan Province (Bernhauer 1938; Hua 2002; Watanabe and Xiao 1994, 1996, 1997, 2000), one from Guizhou Province (Chen et al. 2005a) and two from Shaanxi Province (Chen et al. 2005b).

In 2006, Hu and Tang made a collecting trip to the Hailuogou and the Labahe natural reserves and collected a number of *Lathrobium* specimens. Among them, three new species are recognized and reported herein and a checklist of Chinese *Lathrobium* is provided.

## Material and methods

All specimens were collected from the leaf litter of in broad-leaved forests by sifting. The following abbreviations are used in the text, with all measurements in millimeters:

body length (BL): length of body from the labral anterior margin to the anal apex;

forebody length (FL): length of the clypeal anterior margin to the elytral apex;

head length (HL): length of head from the clypeal anterior margin to the posterior margin of the head;

head width (HW): maximum width of head;

pronotum length (PL): length of pronotum along midline;

pronotum width (PW): maximum width of pronotum;

elytra length (EL): length of elytra from the apex of the scutellum to the elytral posterior margin.

The type material is deposited in the Insect Collection of Shanghai Normal University, Shanghai, China. (SNUC).

## Descriptions

### Lathrobium (Lathrobium) acutissimum

Peng, Li & Zhao sp. n.

urn:lsid:zoobank.org:act:F4955AB5-B251-496B-9802-D3D26E6D39AE

http://species-id.net/wiki/Lathrobium_acutissimum

Figs 1A , 2

#### Type locality.

Labahe Natural Reserve, Sichuan Province, Southwest China

#### Type material (5 ♂♂, 4 ♀♀)

Holotype: ♂, labeled ‘**CHINA:** Sichuan Prov. / Tianquan County / Labahe N. R. / 30°09'N, 102°26'E / 30.vii.2006, alt. 2,000 m / Hu & Tang leg.’. Paratypes: 4 ♂♂, 2 ♀♀, same label data as holotype; 2 ♀♀, same label data, except ‘29.v.2006’.

#### Description.

Measurements and ratios:BL 8.12–10.00, FL 3.78–4.11, HL 1.18–1.26, HW 1.26–1.31, PL 1.52–1.63, PW 1.30–1.41, EL 0.98–1.05, HL/HW 0.93–0.96, HW/PW 0.94–0.97, HL/PL 0.76–0.79, PL/PW 1.16–1.17, EL/PL 0.64–0.67.

**Figure 1.**
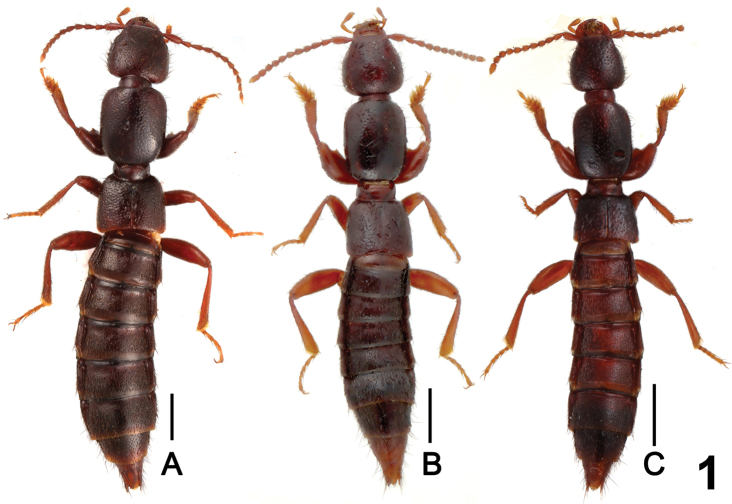
Male habitus of *Lathrobium* spp., **A** *Lathrobium acutissimum* **B** *Lathrobium hailuogouense* **C** *Lathrobium labahense*. Scales: 1.0 mm.

Habitus as in Fig. 1A. Body brown with paler apex, legs brown to light brown, antennae brown to reddish brown.

Head subquadrate (HL/HW 0.93–0.96); punctation coarse and dense; interstices with shallow and netlike microsculpture; eyes small, approximately 1/5–1/4 of length of postocular region in dorsal view.

Pronotum nearly parallel-sided; punctation sparser than that of head; impunctate midline narrow; interstices shining without microsculpture.

Elytra with punctation denser than that of pronotum and well defined; hind wings reduced.

Abdomen with dense punctation; interstices with very shallow, transversely striate microsculpture.

**Figure 2.**
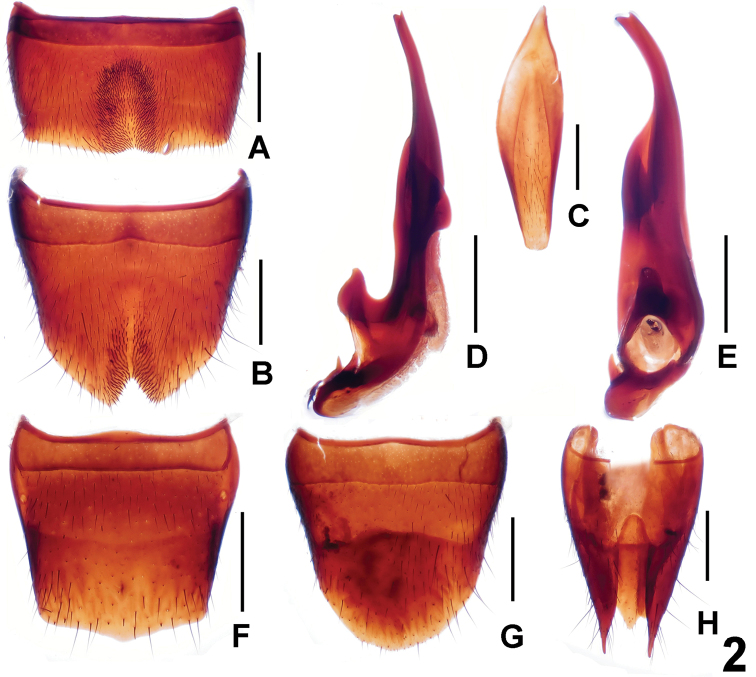
*Lathrobium acutissimum*. **A** male sternite VII **B** male sternite VIII **C** male sternite IX **D** aedeagus in lateral view **E** aedeagus in ventral view **F** female tergite VIII**G** female sternite VIII **H** female tergites IX–X. Scales: 0.5 mm.

Male. Sternite VII (Fig. 2A) with short, darkish setae in U-shaped posterio-median impression; sternite VIII (Fig. 2B) with triangular, symmetrical emargination and short, darkish setae in shallow impression; sternite IX (Fig. 2C) long and nearly symmetrical; aedeagus (Fig. 2D, 2E) with very slender, ventral process.

Female. Posterior margin of tergite VIII (Fig. 2F) somewhat convex; sternite VIII (Fig. 2G) slightly longer than that of male, posterior margin broadly convex; tergite X (Fig. 2H) not acute basally and not reaching anterior margin of tergite IX (Fig. 2H).

#### Distribution.

Southwest China: Sichuan.

#### Etymology.

The specific epithet (Latin, adjective: sharp) alludes to the apical margin of the male sternite IX.

#### Remarks.

It resembles *Lathrobium lijiangense* Watanabe & Xiao, 1997 from Yunnan in having a similar shape of the male sternite VII. The new species can be readily distinguished from these species by the male sternite VIII with a triangular emargination at the apical margin and by the aedeagus with a much longer ventral process. In *Lathrobium lijiangense*, the male sternite VIII has a semi-elliptical emargination at the apical margin and the ventral process of the aedeagus is short.

### Lathrobium (Lathrobium) hailuogouense

Peng, Li & Zhao sp. n.

urn:lsid:zoobank.org:act:9D0986A3-9EFF-43D6-9414-3CD35F1F17FC

http://species-id.net/wiki/Lathrobium_hailuogouense

Figs 1B , 3

#### Type locality.

Hailuogou, Sichuan Province, Southwest China

#### Type material (10 ♂♂, 13 ♀♀)

Holotype: ♂, labeled ‘**CHINA:** Sichuan Prov. / Luding County / Hailuogou / 29°41'N, 102°06'E / 23.vii.2006, alt. 2,200–2,300 m / Hu & Tang leg.’. Paratypes: 4 ♂♂, 4 ♀♀, same label data as holotype; 3 ♂♂, 4 ♀♀, same data, except ‘29°41'N, 102°07'E / 24.vii.2006, alt. 2,800–3,000 m’; 2 ♂♂, 4 ♀♀, same data, except ‘27.vii.2006’; 1 ♀, same data, except ‘28.vii.2006’.

#### Description.

Measurements and ratios:BL 7.23–8.34, FL 3.34–3.72,HL 1.04–1.11, HW 1.05–1.15, PL 1.39–1.48, PW 1.07–1.20, EL 0.93–1.02, HL/HW 0.94–0.99, HW/PW 0.96–0.99, HL/PL 0.75–0.79, PL/PW 1.23–1.30, EL/PL 0.67–0.69.

Habitus as in Fig. 1B. General appearance similar to *Lathrobium acutissimum*, except for somewhat smaller body size and sparser punctation on head and pronotum.

**Figure 3.**
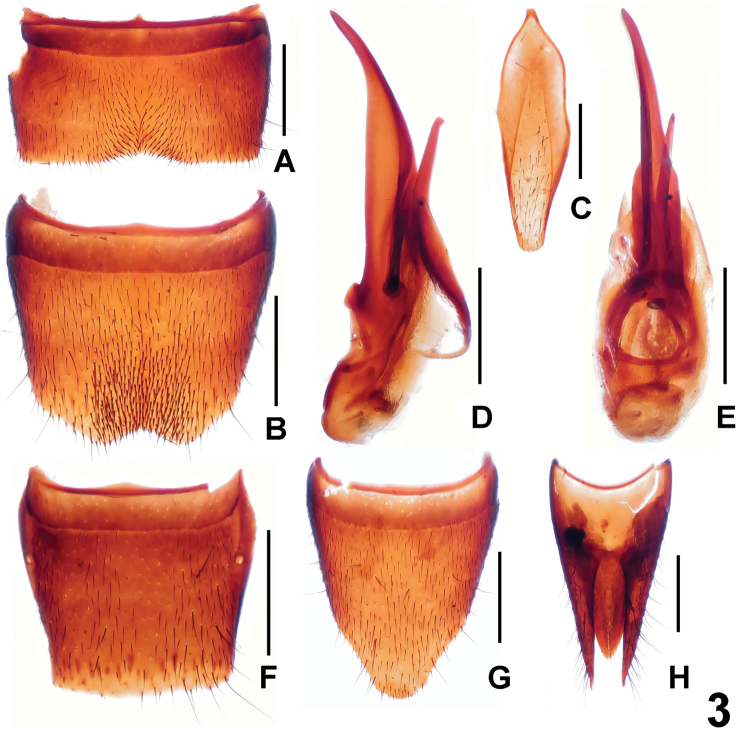
*Lathrobium hailuogouense*. **A** male sternite VII **B** male sternite VIII **C** male sternite IX **D** aedeagus in lateral view **E** aedeagus in ventral view **F** female tergite VIII**G** female sternite VIII **H** female tergites IX–X. Scales: 0.5 mm.

Male. Posterior margin of sternite VII (Fig. 3A) weakly concave; sternite VIII (Fig. 3B) with symmetrical, subtriangular emargination and darkish setae in large, shallow impression; sternite IX (Fig. 3C) asymmetrical; aedeagus (Fig. 3D, 3E) with slender, ventral process and short, dorsal sclerites.

Female. Posterior margin of tergite VIII (Fig. 3F) nearly truncate; sternite VIII (Fig. 3G) much longer than that of male, posterior margin strongly convex; tergite X (Fig. 3H) slender and not reaching anterior margin of tergite IX (Fig. 3H).

#### Distribution.

Southwest China: Sichuan.

#### Etymology.

The specific epithet of this new species is derived from the type locality “Hailuogou”.

#### Remarks.

The new species resembles *Lathrobium zhangi* Watanabe & Xiao, 1997 from Yunnan in having the posterior margin of the male sternite VII weakly concave and a similar shape of the male sternite VIII. The new species can be readily distinguished from these species by the broad ventral process of the aedeagus in lateral view. In *Lathrobium zhangi*, the ventral process of the aedeagus is much narrower in lateral view.

### Lathrobium (Lathrobium) labahense

Peng, Li & Zhao sp. n.

urn:lsid:zoobank.org:act:06462E31-EB5D-4F33-849E-E224427D4CC6

http://species-id.net/wiki/Lathrobium_labahense

Figs 1C , 4

#### Type locality.

Labahe Natural Reserve, Sichuan Province, Southwest China

Holotype: ♂, labeled ‘**CHINA:** Sichuan Prov. / Tianquan County / Labahe N. R. / 30°09'N, 102°28'E / 31.vii.2006, alt. 2,400–2,600 m / Hu & Tang leg.’. Paratypes: 1♀, same label data as holotype.

#### Description.

Measurements and ratios:BL 7.02–7.54, FL 2.95–3.06, HL 0.85–0.94, HW 0.88–0.92, PL 1.22–1.25, PW 0.91–0.93, EL 0.78–0.81, HL/HW 0.97–1.02, HW/PW 0.97–0.99, HL/PL 0.70–0.75, PL/PW 1.34, EL/PL 0.64–0.65.

Habitus as in Fig. 1C. Generally similar to *Lathrobium acutissimum* except for lighter coloration of legs, smaller body size, and somewhat sparser punctation on head and pronotum.

**Figure 4.**
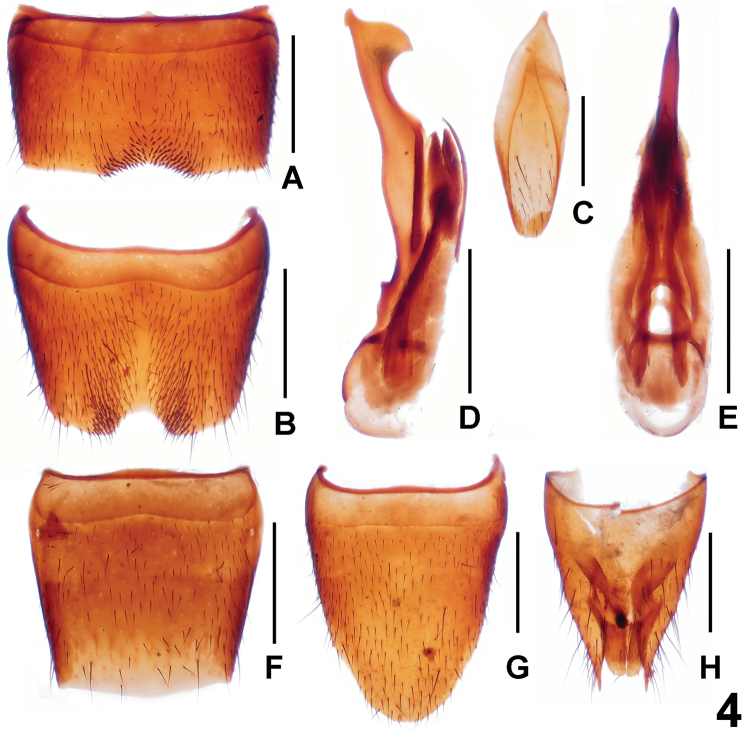
*Lathrobium labahense*. **A** male sternite VII **B** male sternite VIII **C** male sternite IX **D** aedeagus in lateral view **E** aedeagus in ventral view **F** female tergite VIII**G** female sternite VIII **H** female tergites IX–X. Scales: 0.5 mm.

Male. Posterior margin of sternite VII (Fig. 4A) concave and with short, darkish setae; sternite VIII (Fig. 4B) with semicircular, symmetrical emargination and dense, long setae in shallow impression; sternite IX (Fig. 4C) nearly symmetrical; aedeagus (Fig. 4D, 4E) with long, ventral process and thin, dorsal sclerite.

Female. Posterior margin of tergite VIII (Fig. 4F) weakly convex; sternite VIII (Fig. 4G) much longer than that of male, posterior margin strongly convex; tergite IX (Fig. 4H) not separated from tergite X (Fig. 4H).

#### Distribution.

Southwest China: Sichuan.

#### Etymology.

The specific epithet of this new species is derived from the type locality “Labahe”.

#### Remarks.

*Lathrobium labahense* resembles *Lathrobium watanabei* Schülke, 2002 in having a similar shape of the male sternite VIII, but can be readily separated by the aedeagus with a much longer ventral process.

## A checklist of Chinese *Lathrobium*

The checklist is presented in Table 1. The species are arranged alphabetically, and all of them belong to the nominate subgenus. For synonymies see Smetana (2004).

*Lathrobium nomurai* Nakane, 1955 was listed for the China in Smetana (2004). However, this species is apterous and was originally described from Tsuruoka-Do Cave, Kyushu, Japan. Since it is most unlikely that it could be also found in China, we exclude *Lathrobium nomurai* from the list.

The literature references are abbreviated as follows:

A09 Assing (2009)

A10a Assing (2010a)

A10b Assing (2010b)

Aip Assing (in press)

B38 Bernhauer (1938)

C05a Chen et al. (2005a)

C05b Chen et al. (2005b)

HE03 Herman (2003)

HU02 Hua (2002)

K46 Kolenati (1846)

L92 Li (1992)

LC90 Li and Chen (1990)

LC93 Li and Chen (1993)

P12a Peng et al. (2012a)

P12b Peng et al. (2012b)

Ppp Peng et al. (present paper)

R11 Ryvkin (2011)

S62 Scheerpeltz (1962)

SCH02 Schülke (2002)

SCR59 Scriba (1859)

SH74 Sharp (1874)

SH89 Sharp (1889)

SM04 Smetana (2004)

W97 Watanabe (1997)

W98 Watanabe (1998)

W99a Watanabe (1999a)

W99b Watanabe (1999b)

W05 Watanabe (2005)

W11 Watanabe (2011)

WL92 Watanabe and Luo (1992)

WX94 Watanabe and Xiao (1994)

WX96 Watanabe and Xiao (1996)

WX97 Watanabe and Xiao (1997)

WX00 Watanabe and Xiao (2000)

**Table 1.** Checklist of Chinese *Lathrobium*. Footnotes: 1: doubtful record (likely misidentification); 2: communicated by Assing (personal communication).

**Species**	**Distribution in China**	**References**
*Lathrobium acutissimum* sp. n.	Sichuan: Jiajin Shan	Ppp
*Lathrobium ailaoshanense* Watanabe & Xiao, 1997	Yunnan: Ailao Shan	SM04, WX97
*Lathrobium alesi* Assing, 2010	Taiwan: Hsuehshan	A10b
*Lathrobium alishanum* Assing, 2010	Taiwan: Alishan	A10b
*Lathrobium anmaicum* Assing, 2010	Taiwan: Anmashan	A10b
*Lathrobium aokii* Watanabe & Xiao, 2000	Yunnan: Diancang Shan	SM04, WX00
*Lathrobium baihualingense* Watanabe & Xiao, 2000	Yunnan: Gaoligong Shan	SM04, WX00
*Lathrobium baizuorum* Watanabe & Xiao, 2000	Yunnan: Diancang Shan	SM04, WX00
*Lathrobium cooteri* Watanabe, 1999	Zhejiang: Linglong Shan	SM04, W99b
*Lathrobium cylindricum* Bernhauer, 1938	Jiangsu: Chinkiang	HU02, B38, SM04
*Lathrobium dabeiense* Watanabe & Xiao, 1997	Yunnan: Gaoligong Shan	SM04, WX97
*Lathrobium daliense* Watanabe & Xiao, 1994	Yunnan: Diancang Shan	SM04, WX94
*Lathrobium dignum* Sharp, 1874	Hubei, Jiangsu^2^, Liaoning	HU02, L92, LC93, R11, SH74, SM04
*Lathrobium extraculum* Assing, 2010	Taiwan: Peitawushan	A10b
*Lathrobium follitum* Assing, 2010	Taiwan: Peitawushan	A10b
*Lathrobium fulvipenne* Gravenhorst, 1806	Heilongjiang	SM04
*Lathrobium guizhouensis* Chen, Li & Zhao, 2005	Guizhou: Fanjing Shan	C05a
*Lathrobium hailuogouense* sp. n.	Sichuan: Gongga Shan	Ppp
*Lathrobium heteromorphum* Chen, Li & Zhao, 2005	Shaanxi: Taibai Shan	C05b
*Lathrobium houhuanicum* Assing, 2010	Taiwan: Houhuanshan	A10b
*Lathrobium hunanense* Watanabe, 2011	Hunan: Zhangjiacao	W11
*Lathrobium imadatei* Watanabe, 1992	Zhejiang: Wuyanling	SM04, W92
*Lathrobium involutum* Assing, 2010	Taiwan: Hseuhshan	A10b
*Lathrobium ishiianum* Watanabe & Xiao, 2000	Yunnan: Gaoligong Shan	SM04, WX00
*Lathrobium itohi* Watanabe & Xiao, 2000	Yunnan: Gaoligong Shan	SM04, WX00
*Lathrobium jingyuetanicum* Li & Chen, 1990	Jilin: Jingyuetan	L92, LC90, LC93
*Lathrobium jiulongshanense* Peng & Li, 2012	Zhejiang: Jiulongshan	P12b
*Lathrobium jizushanense* Watanabe & Xiao, 1997	Yunnan: Jizu Shan	SM04, WX97
*Lathrobium kishimotoi* Watanabe, 2011	Hunan: Zhangjiacao	W11
*Lathrobium kobense* Sharp, 1874^1^	Jilin?, Hubei?	HU02, LC93, SM04
*Lathrobium labahense* sp. n.	Sichuan: Labahe	Ppp
*Lathrobium lijiangense* Watanabe & Xiao, 1997	Yunnan: Yulongxue Shan	SM04, WX97
*Lathrobium lingae* Peng, Li & Zhao, 2012	Zhejiang: Longwangshan	P12a
*Lathrobium lineatocolle* Scriba, 1859^1^	Jilin?	A09, A10a, K46, LC93, SCR59, SM04
*Lathrobium lobrathiforme* Assing, in press	Yunnan: Gaoligong Shan	Aip
*Lathrobium lobrathioides* Assing, in press	Sichuan: Jinfo Shan	Aip
*Lathrobium longwangshanense* Peng, Li & Zhao, 2012	Zhejiang: Longwangshan	P12a
*Lathrobium miaoershanum* Watanabe, 2011	Guangxi: Maoershan	W11
*Lathrobium monilicorne* Sharp, 1889^1^	Jilin?	L92, LC93, SH89, SM04
*Lathrobium naxii* Watanabe & Xiao, 1996	Yunnan: Yulongxue Shan	SM04, WX96
*Lathrobium nenkaoicum* Assing, 2010	Taiwan: Nenkaoshan	A10b
*Lathrobium pollens* Sharp, 1889^1^	Jilin?	L92, LC93, SM04
*Lathrobium rougemonti* Watanabe, 1999	Zhejiang: West Tianmu Shan	SM04, W99
*Lathrobium semistriatum* Scheerpeltz, 1962	Shandong: Taishan	HU02, S62, SM04
*Lathrobium shaanxiensis* Chen, Li & Zhao, 2005	Shaanxi: Taibai Shan	C05b
*Lathrobium shaolaiense* Watanabe, 1998	Taiwan: Ta-hsüeh Shan	SM04, W98
*Lathrobium sheni* Peng & Li, 2012	Zhejiang: Jiulongshan	P12b
*Lathrobium shuheii* Watanabe & Xiao, 2000	Yunnan: Gaoligong Shan	SM04, WX00
*Lathrobium sinense* Herman, 2003	Jiangsu: Chinkiang, Yunnan?	B38, HE03, HU02, SM04,
*Lathrobium tamurai* Watanabe, 1992	Zhejiang: Wuyanling	SM04, W92
*Lathrobium tarokoense* Assing, 2010	Taiwan: Taroko N. R.	A10b
*Lathrobium tianmushanense* Watanabe, 1999	Zhejiang: West Tianmu Shan	SM04, W99
*Lathrobium tsuifengense* Watanabe, 2005	Taiwan: Tsuifeng	W05
*Lathrobium uncum* Peng, Li & Zhao, 2012	Zhejiang: Longwangshan	P12a
*Lathrobium utriculatum* Assing, 2010	Taiwan: Peinantashan	A10b
*Lathrobium watanabei* Schülke, 2002	Sichuan: Daxue Shan	SCH02
*Lathrobium xiei* Watanabe & Xiao, 2000	Yunnan: Gaoligong Shan	SM04, WX00
*Lathrobium yasutoshii* Watanabe, 2005	Taiwan: Lishan	W05
*Lathrobium yinae* Watanabe & Xiao, 1997	Yunnan: Yulongxue Shan	SM04, WX97
*Lathrobium yunnanum* Watanabe & Xiao, 1994	Yunnan: Laohu Shan	SM04, WX94
*Lathrobium zhangi* Watanabe & Xiao, 1997	Yunnan: Jizu Shan	SM04, WX97
*Lathrobium zhaotiexiongi* Peng, Li & Zhao, 2012	Zhejiang: Jiulongshan, Majian	P12b

## Supplementary Material

XML Treatment for Lathrobium (Lathrobium) acutissimum

XML Treatment for Lathrobium (Lathrobium) hailuogouense

XML Treatment for Lathrobium (Lathrobium) labahense
